# Inhibition of telomerase activity preferentially targets aldehyde dehydrogenase-positive cancer stem-like cells in lung cancer

**DOI:** 10.1186/1476-4598-10-96

**Published:** 2011-08-09

**Authors:** Diego Serrano, Anne-Marie Bleau, Ignacio Fernandez-Garcia, Tamara Fernandez-Marcelo, Pilar Iniesta, Carlos Ortiz-de-Solorzano, Alfonso Calvo

**Affiliations:** 1 Laboratory of Novel Therapeutic Targets, Oncology Division, Center for Applied Medical Research (CIMA), University of Navarra, Pamplona, Spain; 2 Department of Histology and Pathology, University of Navarra, Pamplona, Spain; 3 Cancer Imaging Laboratory, Oncology Division, Center for Applied Medical Research (CIMA), University of Navarra, Pamplona, Spain; 4 Department of Biochemistry and Molecular Biology II, School of Pharmacy, Complutense University, IdISSC, Madrid, Spain

**Keywords:** Lung cancer, ALDH activity, cancer stem cells, telomerase

## Abstract

**Background:**

Mortality rates for advanced lung cancer have not declined for decades, even with the implementation of novel chemotherapeutic regimens or the use of tyrosine kinase inhibitors. Cancer Stem Cells (CSCs) are thought to be responsible for resistance to chemo/radiotherapy. Therefore, targeting CSCs with novel compounds may be an effective approach to reduce lung tumor growth and metastasis. We have isolated and characterized CSCs from non-small cell lung cancer (NSCLC) cell lines and measured their telomerase activity, telomere length, and sensitivity to the novel telomerase inhibitor MST312.

**Results:**

The aldehyde dehydrogenase (ALDH) positive lung cancer cell fraction is enriched in markers of stemness and endowed with stem cell properties. ALDH+ CSCs display longer telomeres than the non-CSC population. Interestingly, MST312 has a strong antiproliferative effect on lung CSCs and induces p21, p27 and apoptosis in the whole tumor population. MST312 acts through activation of the ATM/pH2AX DNA damage pathway (short-term effect) and through decrease in telomere length (long-term effect). Administration of this telomerase inhibitor (40 mg/kg) in the H460 xenograft model results in significant tumor shrinkage (70% reduction, compared to controls). Combination therapy consisting of irradiation (10Gy) plus administration of MST312 did not improve the therapeutic efficacy of the telomerase inhibitor alone. Treatment with MST312 reduces significantly the number of ALDH+ CSCs and their telomeric length *in vivo*.

**Conclusions:**

We conclude that antitelomeric therapy using MST312 mainly targets lung CSCs and may represent a novel approach for effective treatment of lung cancer.

## Background

Each year, lung cancer is responsible for over 200,000 deaths in the USA [1]. Standard treatments include surgical resection, radiotherapy and chemotherapy. Although patients present an initial response to treatment, tumors often relapse leading to a 5-year survival rate of about 15%. Chemotherapeutic drugs most efficiently target the tumor bulk, but a smaller fraction of cells tend to exhibit robust resistance, which has been attributed to the presence of cancer stem cells (CSCs) [2]. The CSC hypothesis has recently received massive attention, particularly because it defines CSCs as the tumor initiating cells [3] with the ability to survive initial treatment and give rise to tumor recurrence and promote metastasis [4].

CSCs have been isolated using a variety of stem cell markers and phenotypes, although their reliability appears to depend on tumor type. In non-small cell lung cancer, CD133 has recently been reported to identify tumor-initiating cells [5] but other studies conducted in various solid tumors demonstrated that CD133 negative cells possess similar tumorigenic activity, suggesting that CD133 is not an optimal marker for the isolation of CSCs [6,7]. The side population (SP) phenotype, conferred by the ability of ABC transporters to efflux the fluorescent Hoechst dye, has also been shown to define cells with stem cell properties in NSCLC cell lines [8]. ABCG2, a stem cell marker of a variety of tissues, proved to be the transporter responsible for the multidrug-resistance phenotype in isolated SP cells [9]. However, Meng *et al.*, demonstrated that up to 45% of cells in NSCLC and SCLC cell lines show tumorigenic potential, regardless of the SP phenotype and CD133 expression [7]. Measurement of aldehyde dehydrogenase (ALDH) activity recently offered a more promising avenue. ALDHs form a group of NAD(P)+ dependent enzymes involved in the oxidation of aldehydes and production of retinoic acid [10] that is thought to participate in cellular differentiation and stem cell self-protection [11]. Normal stem cells were shown to contain higher levels of ALDH activity than their more differentiated progeny [12]. ALDH activity and expression are elevated in several tumor types including brain, breast, liver, colon, pancreas and, more recently, lung [13]. Overall, isolation of ALDH positive cells from these tumors has been shown to enrich for tumor initiating cells [14] with increased proliferation rate, migration and adhesion ability, and more recently with metastatic potential in the case of breast cancer [15].

Telomeres protect chromosomes from degradation, irregular recombination and end-to-end fusions [16]. Telomeres decrease in length with every cell division [17] until they reach a critical size [18]. In normal cells, critically short telomeres are recognized by the DNA damage response (DDR) and cells undergo either senescence or apoptosis [19]. Tumor cells are able to overcome senescence by expressing telomerase, an enzymatic complex that consists of three subunits: the Telomerase Reverse Transcriptase (TERT), the Telomerase RNA Component (TERC) and the dyskerin protein (DKC1) [20]. Telomerase protects telomeres from critical shortening, thus allowing continuous cell division [21], and increased telomerase expression has been commonly found in lung cancer [22]. Therefore, because tumor resistance and recurrence have been attributed to the inability of chemotherapeutic drugs to eliminate CSCs, targeting telomerase function might provide a novel and promising approach to specifically eradicate these cells. After thorough characterization of the lung CSC phenotype, the aim of this study was to determine whether antitelomeric therapy effectively targets the CSC population both *in vitro *and *in vivo*.

## Results

### ALDH activity of lung cancer cell lines correlates with stem cell properties

**Figure 1:**
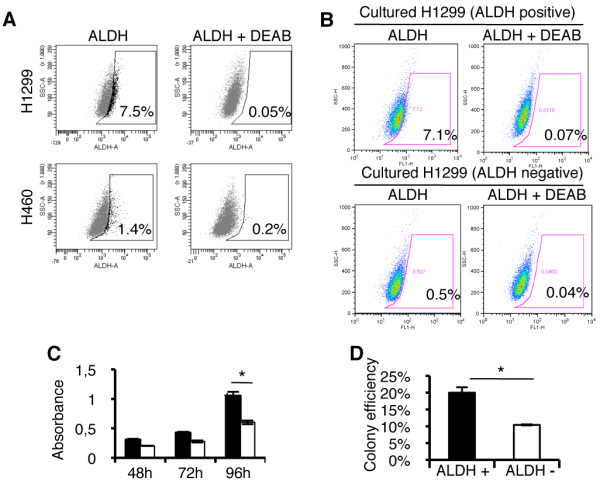
**Characterization of ALDH positive population in human NSCLC cell lines**. *A: *H1299 and H460 cells stained for Aldefluor substrate present a positive population that is blocked by co-incubation with the specific inhibitor DEAB. *B: *Isolated H1299 ALDH+ cells possess self-renewal ability as shown by their capacity to generate both ALDH+ and negative populations after 10 days of culture, while negative cells only give rise to negative cells. *C*: MTT assay demonstrates a greater proliferative rate for ALDH+ cells as compared to the negative population, which reach statistical significance at 96 h. *D: *Isolation of both ALDH positive and negative cells present higher colony formation efficiency in soft agar than their negative counterpart. Data and error bars are presented as mean ± SEM; *: p < 0.05.

We initially analyzed the ALDH+ population of the H1299 and H460 NSCLC cell lines. FACS analysis revealed a clear ALDH+ population that was blocked by co-incubation with the specific enzyme inhibitor DEAB. Average percentages of ALDH+ cells were 7.57 ± 3.64% for H1299 cells, and 1.96 ± 0.83% for H460 cells; representative FACS images are shown in Figure 1A.

To determine if the ALDH+ fraction displayed stem cell properties, we sorted both positive and negative populations from the H1299 cell line and cultured equal numbers of cells in adherent conditions for 10 days. In all cases, we found that only ALDH+ cells were able to generate both the ALDH- and the ALDH+ populations (Figure 1B). Similar findings were observed for H460 cells (see additional file 1, Supplementary figure 1A).

Analysis of the H1299 ALDH+ fraction by MTT revealed an increased proliferative rate of ALDH + cells as compared to the negative fraction, a difference that reached statistical significance at 96 h (p < 0.05) (Figure 1C). To evaluate the *in vitro *malignancy, we looked at the anchorage-independent growth of sorted cells in soft agar. H1299 ALDH+ cells exhibited a two-fold increase in colony formation ability as compared to the negative population (p < 0.05), suggesting a more malignant phenotype (Figure 1D). Overall, these findings confirm the presence of ALDH activity in the CSC population, which correlates with stem cell features.

**Figure 2:**
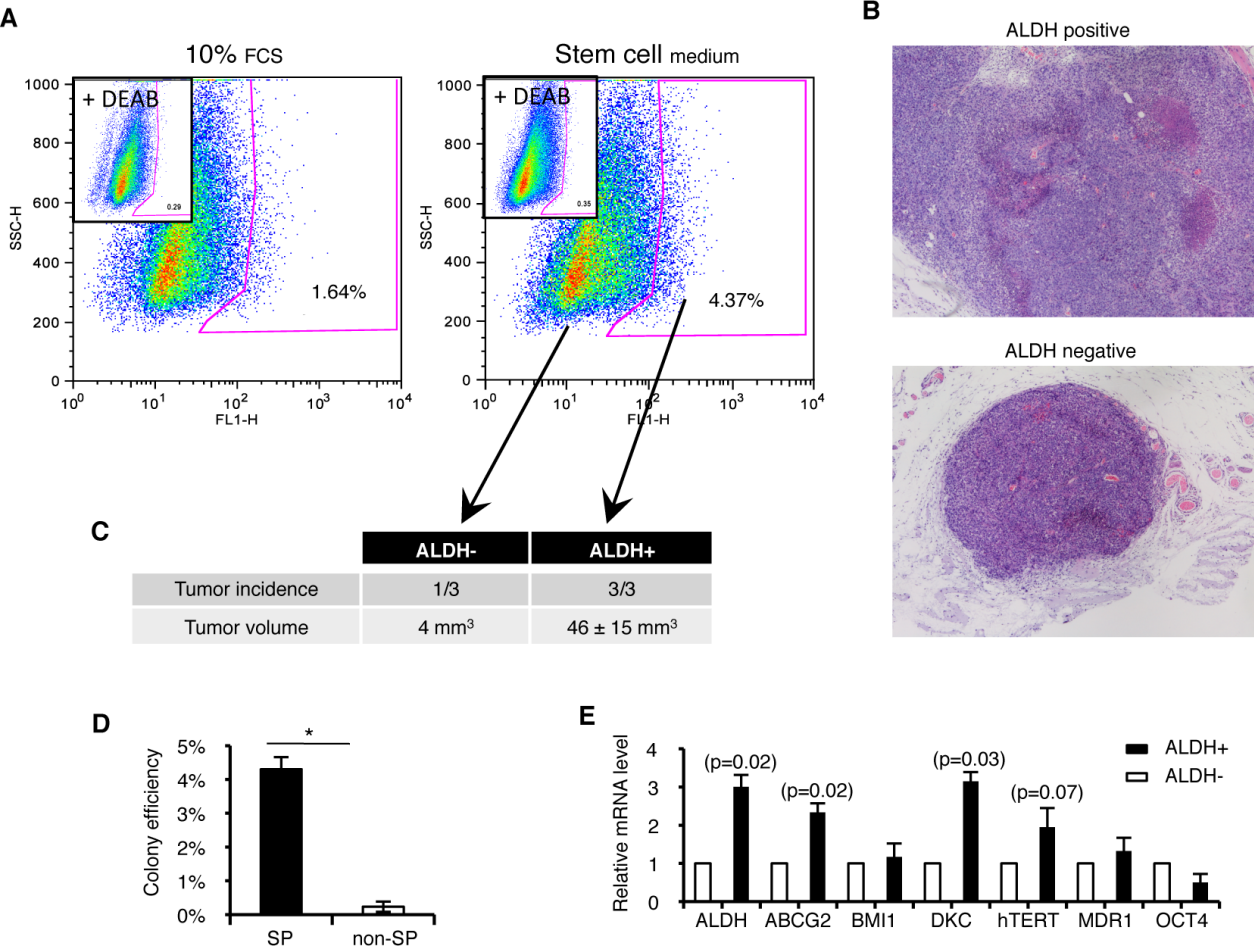
**ALDH positive CSC are endowed with stem cell properties**. *A: *Representative ALDH cytometry from H460 unsorted cells grown in presence of 10% FCS-containing medium or stem cell medium. Percentage of ALDH+ cells in H460 cell line was increased when cultured in stem cell medium. *B: *Hematoxylin eosin staining of tumors engrafted after injection of ten thousand H460 ALDH sorted cells (40X). *C: *Tumor incidence and volume three weeks after cell injection. *D: *Isolation of both SP and non-SP cells present higher colony formation efficiency in soft agar than their negative counterpart. *E: *Q-RT-PCR analysis in sorted H1299 cells confirmed higher expression of ALDH and ABCG2 in ALDH+ cells. These cells also overexpress dyskerin, a component of telomerase subunit. Data and error bars are presented as mean ± SEM; *: p < 0.05, or as indicated.

Finally, we investigated the *in vivo *tumorigenic potential of ALDH cells after injection into mice. As the ALDH+ population isolated from H1299 cells was previously shown to be more tumorigenic than the negative counterpart [23], we focused our study on the H460 cell line. When cultured in stem cell medium (containing EGF, βFGF and B27 supplement), H460 cells displayed a three-fold increase in the percentage of positive cells, further confirming their stem cell properties (Figure 2A). We thus used these cells for sorting and found a greater tumor initiating capacity upon injection of ALDH+ cells. Indeed, 3/3 mice presented a tumor mass with a mean tumor volume of 46 ± 15 mm^3^, while only 1/3 mice developed a small tumor after injection of the negative population (with a tumor volume of 4 mm^3^) (Figure 2B and 2C).

### Phenotypic characterization of the ALDH+ population from NSCLC cells

As the side population (SP) phenotype has been proposed to serve as a stem cell marker in lung cancer cells, we sought to explore more thoroughly the overlap between the SP and ALDH-populations. We identified an SP fraction in H1299 cells of about 2.5%, that was blocked by co-incubation with the ABCG2 inhibitor FTC, confirming that this transporter participates in the SP phenotype of NSCLC. A similar SP fraction was identified in H460 cells (not shown). We next tested the clonogenic capacity of the SP-enriched cell fraction compared to the negative fraction. Indeed, cells within the SP were significantly much more able to form clones than the negative population for both cell lines (Figure 2D and additional file 1, Supplementary figure 1B). To determine if the SP overlapped with the ALDH+ fraction, we performed double staining for Hoechst and ALDH using H1299 cells. Since BAAA is a substrate for ABC transporters and because the Aldefluor buffer contains inhibitors of these pumps, we first sorted SP and non-SP cells and then analyzed both populations by Aldefluor. SP cells were highly positive for ALDH: up to 27.3% of the cells were located in the ALDH+ area, as compared to 3.48% for the non-SP cells (see additional file 1, Supplementary figure 1C).

Q-RT-PCR for genes proposed to represent the CSCs population was further analyzed. As expected, cells sorted for ALDH expressed higher levels of this enzyme (Figure 2E). A two-fold increase in ABCG2 expression in ALDH+ cells as compared to the negative population was noticed. We also found that these cells overexpressed dyskerin, a conserved component of telomerase subunit that is required for telomerase activity (p < 0.05) (Figure 2E). A similar trend was also found for telomerase, although differences did not reach statistical significance. All together, these data confirm the stem cell identity of ALDH+ cells.

### ALDH+ cells present long telomere length but similar telomerase activity compared to ALDH- cells

We first validated the accuracy of the FISH quantification method in NSCLC cell lines by correlating the measurements with those obtained by classical TRF analysis. We quantified telomeres from A549, H1299 and H460 lung cell lines by both TRF and FISH techniques. A statistically significant positive correlation (p < 0.01) between TRF and FISH telomere analysis was observed, allowing to calculate the telomere length based on FISH data (see additional file 1, Supplementary figure 1D-F).

**Figure 3:**
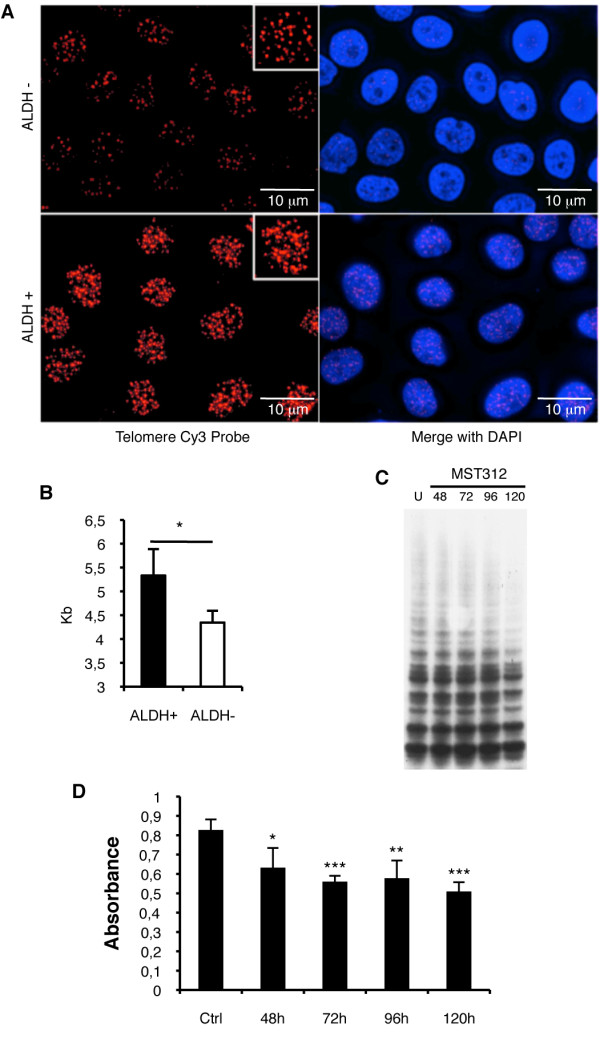
**Lung tumor ALDH+ CSCs present longer telomeres**. *A: *FISH analysis on H460 sorted cells demonstrates that ALDH+ cells possess longer telomeres than the negative counterpart. *B: *Quantification of telomere length confirms that the ALDH+ CSC population has longer telomeres than the negative population. *C and D: *Treatment with MST312 (0.5 μM) for 120 h reduces telomerase activity in the whole population (H1299 cells) as show by PCR analysis (C) and ELISA (D). Data and error bars show mean ± SEM; *: p < 0.05; **: p < 0.01; ***: p < 0.001.

Then, both ALDH+ and ALDH- cells were sorted and plated in equal numbers to quantify telomere length (TL) by FISH analysis. Representative images are shown in Figure 3A. Quantification revealed that the average TL in H460 ALDH+ cells was 5.3 ± 2.7 Kb, which was significantly higher (p < 0.05) than that of ALDH- cells (4.3 ± 2.5 Kb) (Figure 3B). Similar results were obtained for H1299 cells: 17.14 ± 5.5 Kb for ALDH+ cells in contrast to 10.95 ± 4.1 Kb for ALDH- cells (p < 0.001). Our data bring evidence that lung CSCs possess longer telomeres than their differentiated counterparts.

According to the stem cell hypothesis, isolated CSCs should give rise overtime to a large cell population of non-CSCs, while maintaining a similar proportion of CSCs. If this is the case, the average TL in isolated ALDH+ cells should become similar to the average length in the whole population. To address this issue, ALDH+ and ALDH- cells were plated into separate culture slides and TL was measured one and two weeks after plating. Telomeres in the ALDH- population decreased modestly (from 4.7 kb to 4.4 kb) after 3 weeks in culture, compared to TL at day 0 (see additional file 2, Supplementary figure 2A). On the contrary, the ALDH+ fraction, (which after 3 weeks in culture regenerates the whole cell population) was reduced from 5.5 kb to 4.5 kb, not being statistically different from the negative population at this point (see additional file 2, Supplementary figure 2A).

We then used TRAP assay to measure telomerase activity. Both ALDH+ and ALDH- cells showed similar telomerase activity in the two cell lines analyzed (see additional file 2, Supplementary figure 2B). Telomerase activity of H460 cells was higher than that of H1299 cells.

### The novel telomerase inhibitor MST312 preferentially targets ALDH+ CSCs and reduces telomeric length

**Figure 4:**
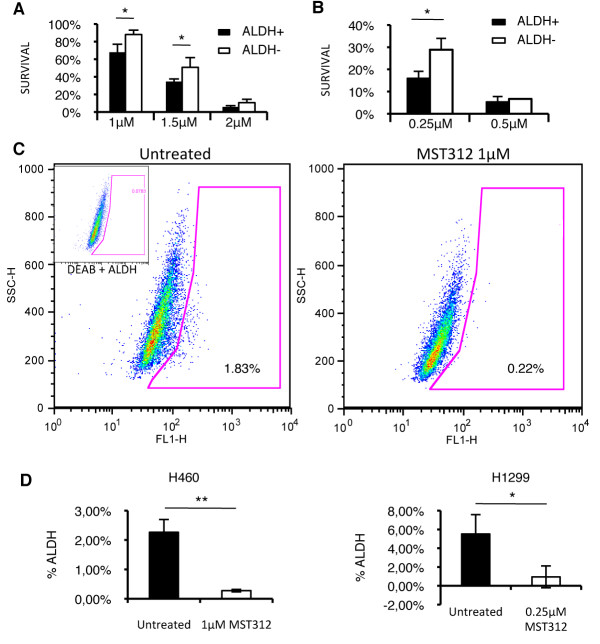
**MST312 effect on both ALDH positive and negative populations**. *A: *96 h incubation with MST312 decreases survival of H460 cells in a dose-dependent manner, with a greater response observed for the ALDH+ fraction. *B: *Similar effects are detected for H1299 cells. *C and D: *MST312 was administered to the whole H460 or H1299 unsorted cell populations for 5 days and ALDH+ cells were quantified by FACS analysis. *D: *FACS quantifications revealed a significant reduction in ALDH+ percentage when cells were treated with MST312 (left: H460 cell line; right: H1299 cell line), thus showing that telomerase inhibition targets the CSC population. Data and error bars are shown as mean ± SEM; *: p < 0.05; **: p < 0.01.

The antitumor effect of the telomerase inhibitor MST312 has been previously demonstrated for different cell lines [24]. We first validated if MST312 also inhibits telomerase activity in H1299 and H460 cells. We found lower telomerase activity 120 h after treatment in H1299 cells (Figure 3C). We also determined by ELISA the telomerase activity; for the untreated H1299 cells, the absorbance was 0.827 ± 0.054 compared to 0.509 ± 0.047 for the H1299 cells treated for 120 h (Figure 3D). We next tested whether antitelomeric therapy would predominantly reduce cell growth in CSCs. To verify this hypothesis, sensitivity to MST312 was tested on ALDH-sorted H460 and H1299 populations. Treatment of sorted ALDH+/- H460 cells induced a dose-dependent decrease in cell survival for both populations; however, ALDH+ cells were more sensitive than negative cells (p < 0.05) (Figure 4A). At each concentration, survival of the ALDH+ fraction was approximately 20 to 25% lower than that observed for the ALDH- population. In the case of H1299 cells, cell growth proved to be highly affected by MST312. At a concentration as low as 0.25 μM, the drug induced an ~80% reduction in cell proliferation, compared to untreated cells. Similarly, sorted ALDH+ H1299 cells were significantly more affected by the treatment than ALDH- cells (Figure 4B). The use of higher doses (0.5 μM or higher) resulted in a dramatic reduction in cell survival (> 90%) and both ALDH+ and ALDH- cells were equally affected (Figure 4B).

To further demonstrate that MST312 altered the number of ALDH+ cells, the whole unsorted cell population was treated for 5 days with the compound and the number of ALDH+ cells was analyzed. Figure 4C shows a representative FACS image in H460 cells demonstrating an eightfold decrease in the number of ALDH+ cells upon MST312 treatment (p < 0.05). Quantifications show a significant decrease in the amount of ALDH+ cells after treatment for both cell lines (Figure 4D).

As shown by our previous experiments, long-term culture of ALDH+ cells repopulates both ALDH+/- cells. To assess whether the telomerase inhibitor reduced global TL, the unsorted whole population of H460 and H1299 cells were cultured for 15 days with a daily low dose of MST312 (1 μM for H460, 0.25 μM for H1299). The average TL was significantly reduced in H460 cells: from 4.8 ± 0.44 Kb to 3.5 ± 0.47 Kb (p < 0.001), and in H1299 cells: from 11.33 ± 1.37 Kb to 9.05 ± 4.54 Kb (p < 0.01).

### MST312 sensitizes ALDH+ CSCs to radiotherapy-mediated cytotoxicity *in vitro*

We next combined antitelomeric therapy with RT (since CSCs are thought to be resistant to irradiation) [25]. The effect of MST312 (1 μM, which reduces cell growth by ~30%), ionizing radiation (3 Gy, which causes ~50% cell growth inhibition), and combination of both treatments was tested on sorted ALDH+/- H460 populations. Irradiation reduced cell viability regardless of ALDH status, while MST312, as previously shown, decreased proliferation of ALDH+ cells compared to the negative population (p < 0.05) (see additional file 2, Supplementary figure 2C). Combination treatment further reduced cell survival especially in the ALDH+ cell population (> 50%, in comparison with a ~20% decrease in the ALDH- fraction). This suggests that MST312 sensitizes the ALDH+ CSCs to suffer a strong cytotoxicity when administered with radiotherapy.

### MST312 treatment blocks cell cycle at the G2/M phase, increases pH2AX, p21 and p27 protein levels, and induces apoptosis

**Figure 5:**
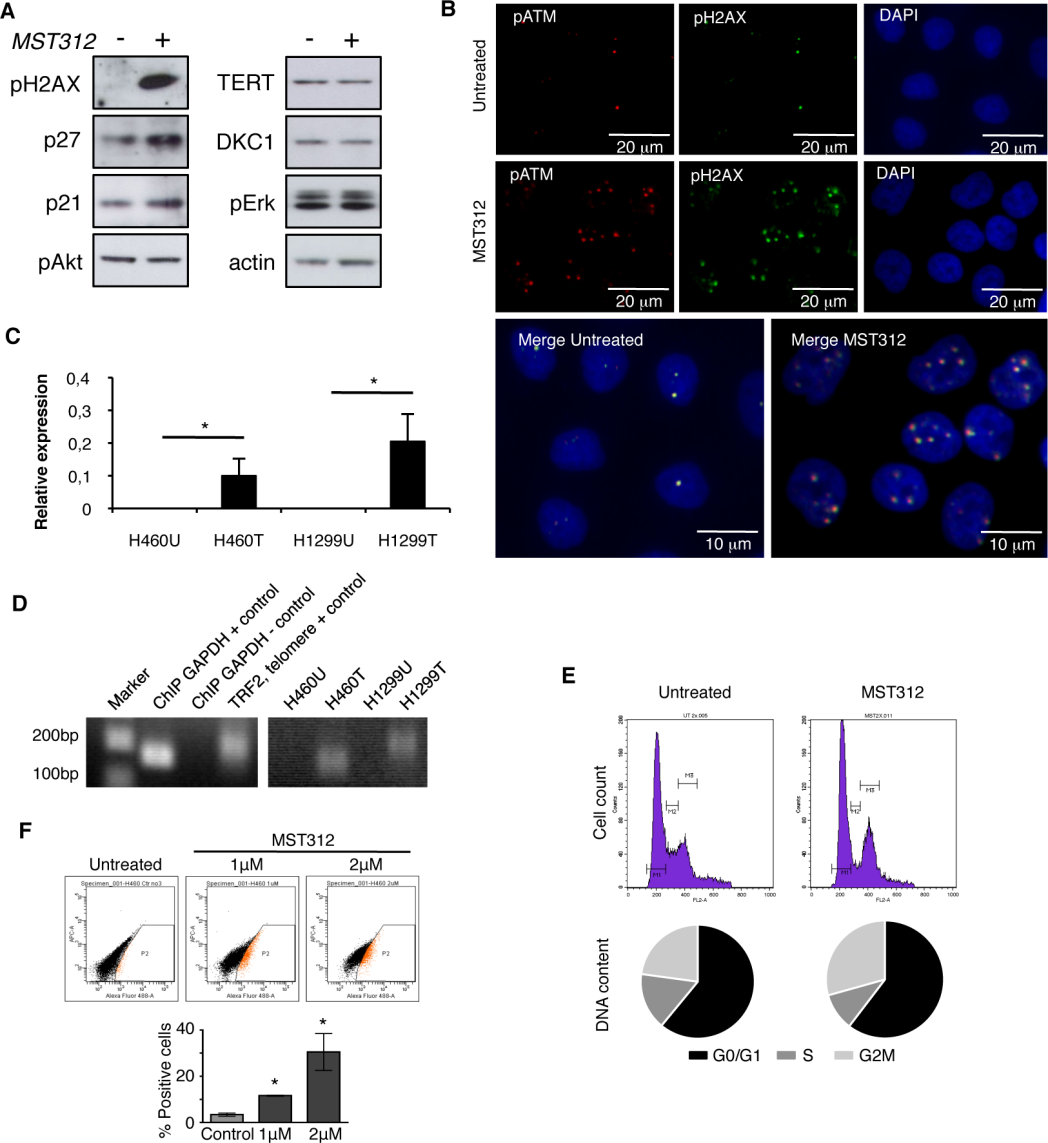
**Regulation of cell cycle distribution and ATM/pH2AX pathway by MST312**. *A: *Western blot analysis shows an increase in pH2AX, p21 and p27 protein levels after 72 h incubation with MST312, while hTERT, dyskerin (DKC1), pAkt and pErk remain unchanged (H460 cells). *B: *Immunofluorescence for pATM (red) and pH2AX (green) in H1299 cells, before and after MST312 treatment. An increase in both pATM and pH2AX (which colocalize) is detected as a result of the exposure to the telomerase inhibitor; DAPI counterstaining, in blue. *C: *Immunoprecipitated chromatin was quantified by QPCR for the detection of telomeric sequences bound to pH2AX. Expression values were normalized with endogenous GAPDH. TRF2 binding to telomeres was used as positive control (U: Untreated, T: MST312 Treated). *D: *Representative immunoprecipitation PCR bands (U: Untreated, T: MST312 Treated). *E: *Cell cycle distribution underlines an arrest in the G2/M phase after treatment, as revealed by an increasing amount of cells in this phase and a decrease in the S phase. *F: *10 days treatment with MST312 induces a dose-dependent increase in active caspase-3, H460 line. Data and error bars are presented as mean ± SEM; *: p < 0.05 compared with untreated control.

To uncover the anti-tumor mechanism responsible for MST312 activity, western blots were performed to measure key downstream telomerase-related signaling proteins. A recent study reported that MST312 induces G2/M cell cycle arrest and acute ATM-pathway-dependent DNA damage in astrocytoma cells [26]. Treatment of H460 cells with MST312 for 72 h resulted in strong increase in the amount of pH2AX (Figure 5A). This result was validated by immunofluorescence. In H1299 untreated cells, low levels of pH2AX and pATM were found and signals for both proteins colocalized (Figure 5B). Administration of MST312 resulted in an increase in pATM and pH2AX signals, which also colocalized (Figure 5B). The same results were observed in H460 line (see additional file 2, Supplementary figure 2D). To further investigate whether MST312 was directly damaging telomere DNA, a ChIP assay was performed to determine if pH2AX binds telomere ends upon treatment. H460 and H1299 cell lines were treated for 72 h with MST312, immunoprecipitated for pH2AX and then telomere sequences were amplified by PCR. In both cell lines, telomere sequence was detected in treated samples but not in untreated cells (Figure 5C and 5D). These results show the activation of pATM/pH2AX pathway within the telomeres by MST312.

We also checked other cell cycle and signal transduction-related proteins. An increase (of about 2-fold) in the amount of p21 and p27 was also found (Figure 5A and additional file 2, Supplementary figure 2E). These changes were accompanied by a significant decrease of cells in the S phase of the cell cycle (from 15.1 ± 0.1% in untreated cells to 9.9 ± 1.1% in treated cells (p = 0.004)), and an increase in the G2/M phase (21.3 ± 0.5%, versus 28.1 ± 0.4% (p = 0.004)) (Figure 5E). Levels of telomerase and dyskerin remained unchanged, confirming that the compound affects telomerase activity rather than its expression. Amounts of pAkt and pErk were not significantly modulated by the treatment. Densitometric quantifications of the bands from 3 different experiments are shown in additional file 2, Supplementary figure 2E. Although the telomerase inhibitor stopped abruptly cell division after a short incubation time (< 3 days in culture), apoptosis was not observed. Nonetheless, 10 days treatment with MST312 induced a dose-dependent increase in active caspase-3 (Figure 5F and additional file 2, Supplementary figure 2F). This demonstrates that apoptosis is a late event that takes place after a cell cycle arrest for several days has occurred.

### MST312 decreases tumor size in mice by inducing apoptosis and reducing proliferation and the number of ALDH+ CSCs

**Figure 6:**
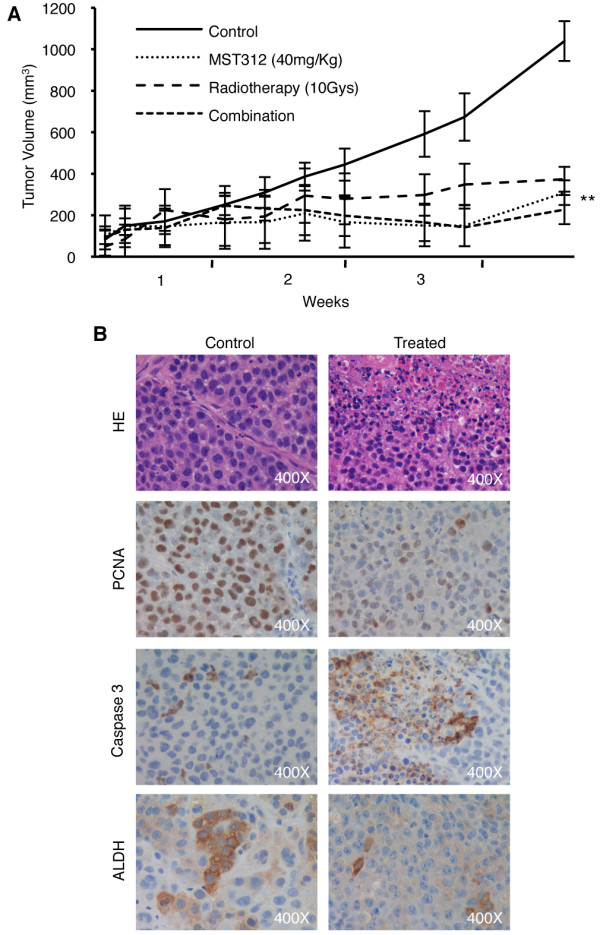
**Telomerase inhibition by MST312 reduces tumor growth *in vivo***. *A: *Tumor growth shows strong reduction in mice that were treated for 3 weeks with MST312 (40 mg/kg, daily). Radiotherapy alone (10 Gys administrated locally at days 3 and 13) produced a similar effect. No significant improvement was obtained by combination of both therapies. *B: *H&E of untreated and MST312-treated mice (upper panel) show tumor cells with pyknotic nuclei upon treatment; immunohistochemistry for PCNA and cleaved-caspase 3 (middle panel) demonstrate a reduction in proliferation and increased apoptosis in mice injected with MST312, compared to controls; immunostaining for ALDH (lower panel) also reveals a reduction in the number of labeled cells after administration of the telomerase inhibitor.

*In vivo *experiments showed that MST312 significantly reduced tumor volume by 70% (p < 0.001) as compared with untreated mice (Figure 6A). Similar effects were observed after RT, which shrank tumor volume by 64% (p < 0.001) compared with controls. Combination treatment resulted in a reduction of tumor volume by 78% (p < 0.001) compared with the control group, but no cumulative benefits were detected *in vivo *compared to therapies alone (Figure 6A).

Hematoxylin-eosin staining showed tumor cells with pyknotic nuclei in MST312-treated mice (Figure 6B). In histological sections, the area fraction corresponding to PCNA-positive immunostaining was 10.87 ± 3.46% for controls and 4.57 ± 2.68% for treated mice (p < 0.001) (see additional file 2, Supplementary figure 2G). Active caspase-3 area fraction was increased by ~10-fold (1.01 ± 0.76% in treated mice compared to 0.18 ± 0.17% in controls, p < 0.001) (see additional file 2, Supplementary figure 2G). Our data demonstrate that administration of MST312 reduces tumor volume by increasing apoptosis and inhibiting proliferation. To ascertain whether MST312 was targeting the ALDH+ CSC population *in vivo*, immunohistochemistry for ALDH (Figure 6B) and image analysis was conducted in tumor sections as well. Quantification of ALDH+ area fraction confirmed a highly significant reduction in mice treated with the telomerase inhibitor as compared to controls (p < 0.001) (see additional file 2, Supplementary figure 2G). At the dose used, no significant weight loss or other signs of toxicity were observed in any of the treated groups. Moreover, the histological appearance of liver, intestine and kidney was normal for mice that received MST312 (see additional file 3, Supplementary figure 3). We also performed FISH analysis in liver sections from both groups of animals and found no difference in telomere length, confirming that treatment seems not to affect normal cells (not shown).

### MST312 targets CSCs *in vivo *and reduces significantly telomeric length

**Figure 7:**
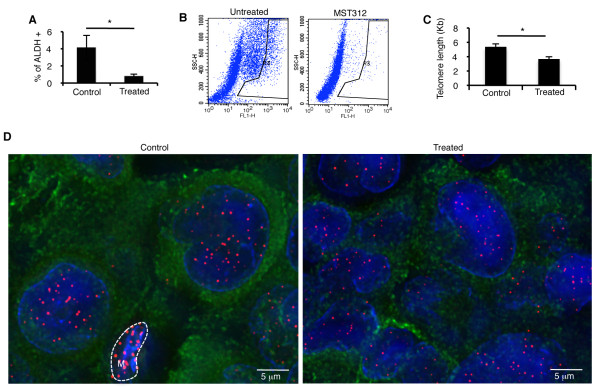
**MST312 targets ALDH+ cells *in vivo *and reduces their telomere length**. *A and B: *FACS analysis and quantification of dissociated tumors confirms, along with the immunhistochemical results, a decrease in ALDH+ cells *in vivo *after treatment with MST312. *C: *Quantification of telomeric length in ALDH+ cells present in tumors from treated and untreated mice. A significant reduction is found in mice administered with the antitelomeric therapy (p < 0.05). ALDH negative cells from the tumor bulk could not be accurately quantified due to massive necrosis. *D: *Representative FICTION image using probes for telomeres (red), immunofluorescence for ALDH (green), and nuclear staining (DAPI, in blue). Images show a lower intensity for telomeres in MST312-treated mice. The "*M" *labeled cell represents a murine fibroblast excluded from the analysis (small nuclei and longer telomeres).

To further verify if MST312 was preferentially targeting ALDH+ cells *in vivo*, tumor cells from untreated and treated mice were isolated and analyzed by Aldefluor. These assays showed a significant reduction in the number of ALDH+ cells upon treatment with MST312 *in vivo*: 4.1 ± 1.4% in untreated mice, versus 0.8 ± 0.21% in treated mice (p < 0.05). Representative FACS images and quantification results are shown in Figure 7A and 7B. All together, these novel results suggest that inhibition of telomerase function directly targets the CSC population both *in vitro *and *in vivo*.

We next quantified TL in the ALDH+/- cell population *in vivo*. To this end, FICTION (Fluorescence Immunophenotyping and Interphase Cytogenetics as a tool for the Investigation of Neoplasms) was performed in order to evaluate simultaneously TL (with the probe specific for telomeres) and ALDH+ cells (by immunofluorescence) (Figure 7C and 7D). Similarly to our *in vitro *observations, results showed that the ALDH+ population had longer telomeres (5.37 ± 0.72 Kb) than ALDH- cells (3.99 ± 0.11 Kb) (p < 0.001) in control mice. We tested whether MST312 was also able to reduce telomere length in xenotransplants. A significant reduction of TL in ALDH+ cells after treatment (3.67 ± 0.54 Kb, p < 0.05) compared to untreated ALDH + cells was found (Figure 7C). Since massive necrotic areas were found in the ALDH- tumor mass, TL could not be accurately measured. Nonetheless, results in the ALDH+ population confirm that MST312 reduces TL *in vivo*.

## Discussion

Cancer stem cells (CSC) are thought to be responsible for the maintenance of the whole cancer cell population and tumor regrowth after therapy. Although a variety of CSC markers have been proposed, some controversy remains over how to characterize the CSC population in different tumor types. In this study, we used the Aldefluor assay (in conjunction with other methods described for CSCs) to isolate and characterize lung CSCs, with the goal of assessing their sensitivity to antitelomeric therapy. We demonstrate here that ALDH+ lung cancer stem-like cells have longer telomeres but similar telomerase activity than the non-CSC fraction, which make them particularly sensitive to the telomerase inhibitor MST312 both *in vitro *and *in vivo*.

Many studies have shown that cancer cell lines (including those of lung cancer) contain functional CSCs with highly tumorigenic potential [27], which serve as models for studying CSC properties. We first confirmed previous findings demonstrating that ALDH activity is a functional marker for NSCLC cell lines [28]. We have thoroughly characterized H460 and H1299 cells showing that they contain a positive ALDH fraction that shares properties of stem cells, including self-renewal ability, high malignant potential *in vitro *as well as increased tumorigenicity *in vivo*. As expected, the ALDH+ population is enriched in markers of stemness, such as ABCG2, and telomerase. Interestingly, the SP of these cells overlaps with the ALDH cell fraction. ALDH+ CSCs display longer telomeres than ALDH- cells, but similar telomerase activity. One study found similar telomere length and telomerase activity in tumor initiating prostate cells (characterized by CD44, integrin α2β1 and CD133 markers) compared to the total cell population [29]. A study conducted in NSCLC demonstrated higher level of hTERT mRNA in isolated SP cells as compared to non-SP cells, suggesting a role for telomerase function in CSCs in the renewal of the tumor population [8].

High expression and activity of telomerase have been reported in more than 80% of tumors, including lung cancer [30]. Most NSCLC and almost all SCLC display substantial high levels of telomerase activity compared to the normal lung; in addition, high hTERT mRNA levels correlate with poor prognosis and tumor recurrence [30]. At the initiation of lung carcinogenesis, a progressive reduction in telomere length has been reported, preceding TRF1/2 overexpression and telomere elongation in established tumors, as a result of telomerase overexpression [22]. In lung cancer, the predictive value of telomere erosion and its relationship with genetic instability are issues still needed to be analyzed in large cohorts of patients. Based on all these data, antitelomeric therapy may be an appropriate approach to treat lung cancer.

Different strategies targeting telomerase are currently being tested in cancer, albeit few studies have analyzed whether these therapies are more effective in CSCs. These include nucleic acid-based methods to inhibit hTERT and hTR, immunotherapy, G-quadruplex ligands, and hTERT-targeting drugs [31]. The most clinically advanced compound is GRN163L (Imetelstat, Geron Corporation), which is currently in phase I clinical trials for the treatment of NSCLC and breast cancer [32]. GRN163L is a synthetic oligonucleotide complementary to the template region of hTR. This drug has been shown to reduce NSCLC and breast cancer growth in xenograft models [33,34].

MST312 is a small molecule that has emerged as a promising candidate for antitelomeric therapy [24]. Previous studies in astrocytoma have shown that this telomerase inhibitor acts through two different mechanisms, depending on the time of exposure [26]. Short-term administration of the drug leads to an acute effect, with ATM-dependent G2/M cell cycle arrest, DNA damage, and reduced cell viability. This effect, which occurs within 72 h after treatment, is not mediated by telomere erosion. Long-term effects using sub-cytostatic doses of MST312, which requires administration of the compound for more than 1.5 months, leads to a significant telomere shortening [26].

Telomere DNA damage during the acute effects suggests that the telomerase complex may uncouple from the DNA, being then recognized by the DNA-double strand damage machinery and activation of the ATM pathway. Increased pH2AX, which has been found in our study and in astrocytoma [26], has been involved in cell cycle arrest as a result of ATM activation. Importantly, we have also demonstrated here that pH2AX binds to the telomeric DNA as a consequence of MST312 administration. Analysis of downstream signaling cascades in the present study showed increased levels of p21 and p27. This correlates with the observation of a cytostatic effect of MST312 during the first days of treatment, without apoptosis induction. Massive apoptosis was detected *in vitro *10 days after exposure to the drug and this result was also corroborated *in vivo *in tumor-bearing mice at the end of the experiment. These data confirm the acute effect of MST312 on lung cancer cells involving the ATM/pH2AX DNA-damage pathway, leading cells to cell cycle arrest and, finally, to apoptosis. Interestingly, Imetelstat (GRN163L), antisense hTERT inhibition and G-cuadruplex ligands exert short-term effects as well (independent of critical telomere erosion), resulting in cell growth inhibition and dramatic changes in cell morphology [35-37]. This suggests novel mechanisms of antitelomeric therapy (unexplored yet) unrelated to telomere attrition that will produce cancer cell derange.

Telomeric length might determine sensitivity to MST312, rather than telomeric activity. According to our results, cells with longer telomeres (H1299 > H460, and CSCs > non-CSCs) are more sensitive to MST312-mediated cell growth inhibition. These findings fit with previous observations, which demonstrated that telomere shortening attenuates the effect of telomerase inhibition by MST312 [38]. Although this hypothesis should be confirmed in future studies using a larger variety of cell lines, it is possible that cells with longer telomeres are more prone to suffer DNA damage because of the telomerase-DNA uncoupling. If confirmed, telomere length may also be an indicator of MST312 response.

We demonstrate here for the first time that the novel telomerase inhibitor MST312 has a potent anti-tumor growth activity on NSCLC *in vivo*, with more than 70% tumor shrinkage using 40 mg/kg daily i.p. injections. Similar tumor reduction (~70%) has been found for human glioblastoma cells xenografted into mice, daily administered with 30 mg/kg Imetelstat [29]. Histologically, tumors from treated mice in our experiment showed large necrotic regions, significantly lower numbers of proliferating cells, and increased number of apoptotic cells. Importantly, a dramatic reduction of the CSC population was also detected at the end of the *in vivo *treatment, as shown both by FACS analysis for ALDH after removal of the tumor, and by immunohistochemistry for ALDH. This is accompanied by a significant reduction of the telomeric length elicited by the drug. These results clearly show that MST312 targets preferentially CSCs *in vivo *and that ALDH expression might be considered as a biomarker of response.

## Conclusions

In summary, we demonstrate herein that the ALDH+ lung cancer stem-like cell population has longer telomeres than non-CSCs and are highly sensitive to the novel telomerase inhibitor MST312 *in vitro *and *in vivo*, suggesting that this compound would mainly target the lung CSC population. We also demonstrate that short-term administration of MST312 switches on the ATM/pH2AX pathway and long-term effects are typically characterized (as expected) by telomere length shortening. Our data support the possible use of MST312 in clinical settings against lung cancer.

## Materials and methods

### Cell culture, proliferation and soft agar colony formation assay

The human NSCLC cell lines H460 and H1299 were obtained from ATCC (Manassas, USA). Cell lines were grown in RPMI (Lonza, Switzerland) supplemented with 10% fetal bovine serum (FBS), 100 unit/ml penicillin G and 100 μg/ml streptomycin in a 37°C humidified 5% CO_2 _incubator. For spheres culture, cells (5 × 10^4 ^cells/mL) were seeded in ultralow attachment dishes (Corning, USA) and grown in serum-free DMEM/F12 medium (Lonza) supplemented with hormone mixture B27 (Gibco, USA), 20 ng/mL EGF (Sigma-Aldrich, USA), 10 ng/mL bFGF (Invitrogen, USA) and 1 μg/mL heparin. Fresh medium was added to the culture every 48 h or 72 h. Spheres were dissociated by incubating them for 5 min with StemPro^® ^Accutase^® ^Cell Dissociation Reagent (Invitrogen).

Cell viability was determined by MTT (3-(4,5-Dimethylthiazol-2-yl)-2,5-diphenyltetrazolium bromide, Roche, Switzerland) assay. ALDH+ and ALDH- cells were seeded in 96-well culture plates (1 × 10^4 ^per well) in 100 μL of medium. 48, 72 and 96 h later, 10 μL of MTT (2.5 mg/ml) was added and cells were further incubated for 4 h, followed by 100 μL of solubilization buffer overnight. Spectrophotometric absorbance was measured at 570 nm.

Soft agar assay for colony formation was performed by seeding sorted cells at 10^3 ^cells into 0.3% agar containing RPMI and 10% FBS over 2-ml base layers (0.6% agar). After 10 days in culture, the number of colonies was assessed by staining with 500 μL 10 mg/mL MTT solution. After incubation for 4 hours, 500 μL DMSO were added, the plates were scanned, and the cell colonies were counted.

### ALDH staining

ALDH staining was performed with the ALDEFLUOR kit (Stem Cell Technologies, Canada) according to the manufacturer's instructions. Briefly, cells were suspended at 1 × 10^6 ^cells/ml in Aldefluor assay buffer containing ALDH substrate (BAAA, BODIPY^® ^aminoacetaldehyde, 1 mmol/l), with or without the specific ALDH inhibitor diethylaminobenzaldehyde (DEAB) (1 mmol/l) for 30 min. DEAB serves as internal negative control for each individual experiment, and allows to distinguish between ALDH-bright (ALDHbr) cells and cells with low ALDH activity (ALDHlow). Analysis and sorting were conducted on a FACSAria IIu (BD, USA): Aldefluor was excited at 488 nm and fluorescence emission was detected at 530/30. Dead cells were excluded by gating on forward and side scatter and eliminating the PI-positive population. The data were analyzed by Cell Quest Pro and *FlowJo *(Ashland, USA).

### Side population analysis

Cells were preincubated at 37°C for 30 min with or without 5 μM fumitremorgin C (Sigma-Aldrich, USA) and incubated for 90 min at 37°C with 5 μg/ml Hoechst 33342 (Sigma-Aldrich). Cells were then kept on ice for 10 min, washed, and sorted with a FACSAria IIu (BD, USA). Hoechst dye was excited at 407 nm by violet laser and its dual wavelengths were detected using 450/40 (Hoechst 33342-Blue) and 620 (Hoechst 33342-Red) filters. Dead cells were excluded as described above.

### Cell cycle analysis

For cell cycle distribution analysis, adherent and floating cells were fixed in 4% paraformaldehyde for 5 min on ice, immersed in 70% ethanol and kept at -20°C for 30 min or until analysis. Cells were rehydrated in PBS, treated with RNase A (500 μg/ml) (Qiagen, Germany) and stained with propidium iodide (50 μg/ml).

### Quantification of mRNA by Q-RT-PCR Analysis

Purified RNA was obtained with the Qiagen RNA isolation kit and cDNA was synthesized with the SuperScript II First-Strand Synthesis System (Invitrogen). The PCR amplification mixture contained cDNA, SYBR Green I Master Mix buffer (Applied Biosystems), and forward and reverse primers (20 nM each). Primers for amplification are shown in Table 1. Real time PCR was carried out with the 7300 RT-PCR system (Applied Biosystems, USA). Every assay was performed in triplicate and all experiments included analysis of GAPDH mRNA levels as internal standard. Relative expression was determined by the Ct method and levels were expressed as percentage relative to the GAPDH mRNA levels.

**Table 1:** List and sequence of primers used in this manuscript for qPCR amplification

Gene	Sequence (5'- > 3')
**ALDH**	s TCCTGGTTATGGGCCTACAG
	as CTGGCCCTGGTGGTAGAATA
**ABCG2**	s CACCTTATTGGCCTCAGGAA
	as CCTGCTTGGAAGGCTCTATG
**BMI1**	s ATGCAGCTCATCCTTCTGCT
	as GCATCACAGTCATTGCTGCT
**DKC**	s TAATGTTGGCCCCATAGCAG
	as CACATGGTGACAATGCATGA
**hTERT**	s CGTGGTTTCTGTGTGGTGTC
	as CCTTGTCGCCTGAGGAGTAG
**MDR1**	s GCTCCTGACTATGCCAAAGC
	as TCTTCACCTCCAGGCTCAGT
**OCT4**	s ACATCAAAGCTCTGCAGAAAGAACT
	as CTGAATACCTTCCCAAATAGAACCC

### Western Blot

Proteins were extracted in RIPA buffer and concentrations were measured by the bicinchoninic acid method (Pierce, USA). Total extracts were electrophoretically fractionated on 10 or 12% Bis-Tris polyacrylamide gels (Invitrogen) and transferred to a 0.45 μm nitrocellulose membrane. Membranes were blocked for 1 h with 5% nonfat dry milk in TBS-Tween and incubated overnight at 4°C with the following primary antibodies: pH2AX, p27, pAkt, pErk1/2 (all of them from Cell Signaling, diluted 1:1000), p21 (Dako, 1:1000), telomerase (Novocastra, 1:1000), dyskerin (Sigma, 1:1000), and β-actin (Sigma, 1:5000). Membranes were washed and incubated with peroxidase-labeled secondary antibodies at room temperature for 1 h. Immunoreactive bands were detected with the Lumi-Light Western Blotting Kit (Roche, Switzerland) and quantified with ImageJ (NIH, USA).

### Chromatin immuno-precipitation assay

ChIP (Chromatin Immuno-Precipitation) assay was performed in both H460 and H1299 cell lines treated with 1 μM and 0.25 μM MST312 respectively, using EZ ChIP kit (Millipore, USA) according to manufacturer's instructions. After cross-linking, cells were lysed and sonicated (15 seconds for 3 times). Sonicated lysates were centrifuged at 14,000 rpm at 4°C for 15 minutes and the cleared chromatin (100 μg) was immunoprecipitated with 5 μg of anti-pH2AX antibody (Cell Signaling, USA) and incubated overnight at 4°C with rotation. TRF2 (Telomeric Repeat-binding Factor 2) antibody (Santa Cruz, USA) was used as positive control for the telomere immuno-precipitation. Quantitative PCR was performed for the detection of telomeric sequence, whose primer sequences were (5'→3'): **tel1**, GGTTTTTGAGGGTGAGGGTGAGGGTGAGGGTGAGGGT and **tel2**, TCCCGACTATCCCTATCCCTATCCCTATCCCTATCCCTA, as previously described [39]. Finally, PCR products were analyzed by agarose gel electrophoresis.

### Telomerase activity assay

Telomerase activity was measured using the TeloTAGGG Telomerase PCR ELISA^PLUS ^TRAP (Telomere Repeat Amplification Protocol, Roche) kit, according to the manufacturer's instructions. The kit includes specific telomere primers bound to biotin, which allows measuring the PCR products (and the telomerase activity) by ELISA. Briefly, 1 μg proteins were loaded into the PCR mix. As positive control, we used an extract of the telomerase-positive cell line 293, and negative controls were prepared in each case by treating cell extracts with DNase-free RNase. To avoid the effect of Taq polymerase inhibitors present in the extracts, we estimated the activity of telomerase by serial dilutions of each sample [40]. Then, PCR reactions were performed using standard conditions and PCR products were run through acrylamide gel electrophoresis at 250 V for 3 hours. The acrylamide gel was stained with SYBR green I (Invitrogen).

### Telomere restriction fragment (TRF) analysis

Telomere length analysis by southern blot was performed with Telo-TAGGG Telomere Restriction Fragment kit (Roche) according to manufacturer's instructions. Briefly, 2 μg of nucleic DNA from each sample were enzymatically digested and run through an electrophoresis gel. Then, the gel was transferred overnight and hybridized using the telomere probe provided by the kit. Luminescence emitted by the probe was detected by exposition to Hyperfilm ECL film (Amersham Bioscience, USA). Finally, the film was scanned and analyzed using ImageJ.

### FISH analysis

To perform FISH analysis for telomeres, cells or tissues were fixed and antigen retrieval using citrate buffer (2.1 g/L, pH 6) and heating (9 min at 95°C followed by 10 min at 85°C), followed by 30 min incubation at room temperature with Tris Buffer Saline with 0.05% Tween (TBS-T) and 5% Triton was used. To block unspecific labelling, samples were incubated with 2% BSA for 30 min, dehydrated in alcohols, dried and incubated with the Telomere PNA-Cy3 probe (Dako, Denmark). We proceeded by co-denaturing both genomic DNA and probes for 5 min on a hot plate at 80°C followed by overnight hybridization at 37°C in a humidified chamber. DAPI was used as a nuclear counterstain.

### Animal model and treatment with the telomerase inhibitor MST312

For the evaluation of the tumor initiating capability of CSC, six-week-old NSG (NOD SCID IL2Rγ mice from The Jackson Laboratory, USA) were used. Mice were maintained in a pathogen-free environment and handled according to our institutional guidelines established for animal care and use. Ten thousand ALDH positive or negative sorted H460 cells were injected subcutaneously into the flanks of the mice in a 1:1 PBS/Matrigel solution (BD). Tumor volume was measured after 3 weeks according to the formula: V = length × (width)^2^/2.

Eight-week-old male athymic mice (Harlan, Spain) were used to test the treatment efficacy of telomerase inhibition. 4 × 10^5 ^H460 cells were injected subcutaneously into the flanks of the mice, and animals were randomly classified in 4 groups: controls (no treatment), MST312-treated, radiotherapy-treated (RT), and the combination MST312+RT. The therapeutic regimen for MST312 consisted of daily i.p. injections (40 mg/kg) for 25 days, starting when tumors reached ~100 mm^3 ^in volume. For RT, two doses of 10 Gys were locally applied on the tumor at days 3 and 13 using a Primus Linear Accelerator (Siemens, Germany). The same doses and administration than those used for single therapies were used for combination assays. Tumor volume was periodically measured and mice were monitored carefully for symptoms of toxicity. All mice were sacrifized 25 days after cell injection and tissues (tumor, liver, intestine and kidneys) were kept for immunohistochemical analysis.

### Tissue dissociation and analysis of ALDH+ cells

Immediately after surgical resection, tumors were minced and digested with Collagenase/Hyaluronidase solution (Stem Cell Technologies) for 4 h at 37°C. Cells were then cultured for 5 h and the ALDH+ population was analyzed. To avoid detection of murine cells we used an anti-mouse cytokeratin (H2Kd) PE-conjugated antibody (BD). Tumor-derived cells were incubated with 1:250 H2Kd antibody in PBS for 15 min at 37°C. Samples were then analyzed by FACS to quantify the ALDH+ population, as described above.

### Histology, immunohistochemistry, immunofluorescence

Tissues were fixed in 10% formalin, embedded in paraffin and sectioned. Slides were stained with Hematoxylin & Eosin. For immunohistochemistry, sections were deparaffinized and incubated for 10 min with 3.3% H_2_O_2 _in water to block endogenous peroxidase. Antigen retrieval was used for the detection of caspase-3, PCNA and ALDH. Sections were incubated with 5% normal goat serum in TBS for 30 min at room temperature. Dilution of primary antibodies was as follows: 1:400 for human anti-PCNA (Dako), 1:200 for human anti-ALDH (BD)[41] and 1:200 for human anti-active caspase-3 (Cell signaling). The EnVision (Dako) system was used to detect the bound antibodies and Harris hematoxylin was utilized for counterstaining.

For immunofluorescence, cells or tissues were fixed, processed according to standard procedures and incubated with an anti-ALDH antibody (BD, 1:50), ATM (Novus Biological, 1:400) and pH2AX (Cell Signaling, 1:50). Secondary antibodies conjugated with 488 Alexa dye (for the anti-ALDH and anti-ATM antibodies), or 594 Alexa dye (for the anti-pH2AX antibody) were applied, and DAPI (Biotium Inc, USA) was used for nuclear counterstaining. Slides were mounted with Slow Fade (Invitrogen) and kept at 4°C.

### Image analysis and quantification

For quantification of immunohistochemistry, 60 random images (x200) per experimental group were captured with a Nikon microscope Y-TSH (Japan). The stained areas were quantified with the ImageJ software and data were expressed as positive immunostained area with respect to reference area.

For quantification of immunofluorescence, thirty plane image stacks were captured 0.29 μm apart. Images were acquired with a motorized Axioplan 2ie (Zeiss, Germany) epifluorescence microscope Plan Apochromat. Images were recorded using a CCD Photometrics monochrome camera (Photometrics^® ^CoolSNAP™*cf*, Microimaging Application Group, USA). The Metamorph^© ^software (Molecular Devices, Canada) was used to control the image acquisition.

Deconvolution, linear unmixing and image segmentation were applied following previously published protocols in order to reduce the background signal and improve quantification accuracy [42]. Briefly, software-based image deconvolution was applied to obtain 3D quasi-confocal sectioning from the wide field image stacks. The model of the Point-Spread Function (PSF) was experimentally calculated for each channel using the TetraSpeck™ Fluorescent Microspheres (Molecular Probes, USA) kit. Deconvolution was performed with the Huygens software (Scientific Volume Imaging BV, The Netherlands). Cross-talk between image channels was estimated and eliminated using a Non Negative Least Squares (NNLS) algorithm [43].

Telomere length was estimated as previously described [42]. A total of 100 nuclei per mice (n = 4) were analyzed. Data are expressed as average of telomeric length.

### Statistical Analysis

Results were analyzed with PRISM 4 for Macintosh (Graphpad software Inc, USA). Comparison between two groups was done with Student's t test and the differences between numerous treatments were evaluated with the analysis of covariance (ANCOVA). P values < 0.05 were considered statistically significant. For tumor volume curves in mice, the non-parametrical Mann Whitney U test was used. For correlation tests, we applied the Pearson correlation coefficient and the F and t coefficients for statistical linear regressions.

## Competing interests

The authors declare that they have no competing interests.

## Authors' contributions

DS, AMB, IFG, COS and AC participated in the experimental design. DS and AMB carried out the majority of the experiments, including CSC characterization, QRT-PCRs, western blots, immunofluorescence/immunohistochemistry, and work related to quantification of telomere length. Both authors wrote most of the manuscript. IFG contributed to quantification of telomere length and its relationship with TRF, and with image analysis procedures. TF and PI carried out the quantification of telomerase activity. COS and AC supervised the progress of the experiments and discussed results. All authors read and edited the manuscript.

## Supplementary Material

Additional file 1 **Supplementary figure 1**. *A: *Demonstration of the self-renewal capacity of ALDH+ H460 cells. Isolated ALDH+ cells give rise to both ALDH+ and ALDH- populations, while negative cells are not able to produce ALDH+ cells. *B: *Sorted H460 SP has significantly higher clonogenic capacity than non-SP cells. *C: *Demonstration that the H1299 SP overlaps with the ALDH+ populations. 27.3% of the SP corresponded to ALDH+ cells, whereas only 3.48% of non-SP were ALDH+. *D: *Representative FISH images from H460, A549 and H1299 unsorted cell lines used for the telomere quantification. *E: *Telomere length analysis by TRF; left, DNA ladder; right, H460, A549 and H1299 genomic DNA digested and hybridized with a telomeric specific probe. *F: *Correlation graph demonstrating the accuracy of extrapolation between image arbitrary units (FISH signals) and telomere length measured by TRF. ***: p < 0.001. Click here for file

Additional file 2 **Supplementary figure 2**. *A: *ALDH+ and ALDH- cells were plated into separate culture slides and telomeric length was measured one and two weeks after plating. Telomeres in the ALDH+ population reached a similar length than that found in the negative fraction. This is likely due to the generation by ALDH+ cells of a majority of ALDH- population overtime, which supports the self-renewal capacity of these cells. *B: *Telomerase activity in both ALDH+/- cells. *C: *MST312 sensitizes ALDH+ cells to radiotherapy-induced cytotoxicity *in vitro*. Radiotherapy (RAD) alone causes a comparable decrease in cell survival of both ALDH positive and negative populations in H460 cells. Administration of MST312 sensitizes cells to irradiation-dependent cytotoxicity. *D: *Immunofluorescence for pATM (red) and pH2AX (green) in H460 cells, before and after MST312 treatment. An increase in both pATM and pH2AX (which co-localize) is detected as a result of the exposure to the telomerase inhibitor. *E: *Densitometric quantification of the bands from western blots of proteins related to cell cycle and DNA damage signaling cascades in untreated and MST312-treated cells. Three western blot analyses were quantified by densitometry; β-actin was used as housekeeping protein and data are presented in fold changes compared to the untreated group. Increase in pH2AX, p21 and p27 protein levels after 72 h incubation with MST312 was observed. No changes were found for hTERT, dyskerin (DKC1), pAkt and pErk. *F: *10 days treatment with MST312 induces a dose-dependent increase in active caspase-3, H1299 cell line. *G: *Quantification of PCNA, cleaved-caspase 3 and ALDH staining by image analysis in tumor sections. *: p < 0.05; **: p < 0.01; ***: p < 0.001 compared with control. Click here for file

Additional file 3 **Supplementary figure 3**. Histological appearance of liver, intestine and kidney in mice that received MST312 (treated) in comparison to controls. No significant changes are appreciated between both groups. Click here for file
