# Supplementary figures and images for: Inhibition of telomerase activity preferentially targets aldehyde dehydrogenase-positive cancer stem-like cells in lung cancer
Source: Mol Cancer. 2011 Aug 9;10:96. doi: 10.1186/1476-4598-10-96 (PMC3199900; doi:10.1186/1476-4598-10-96)

# Supplementary 1

*Serrano et al.*

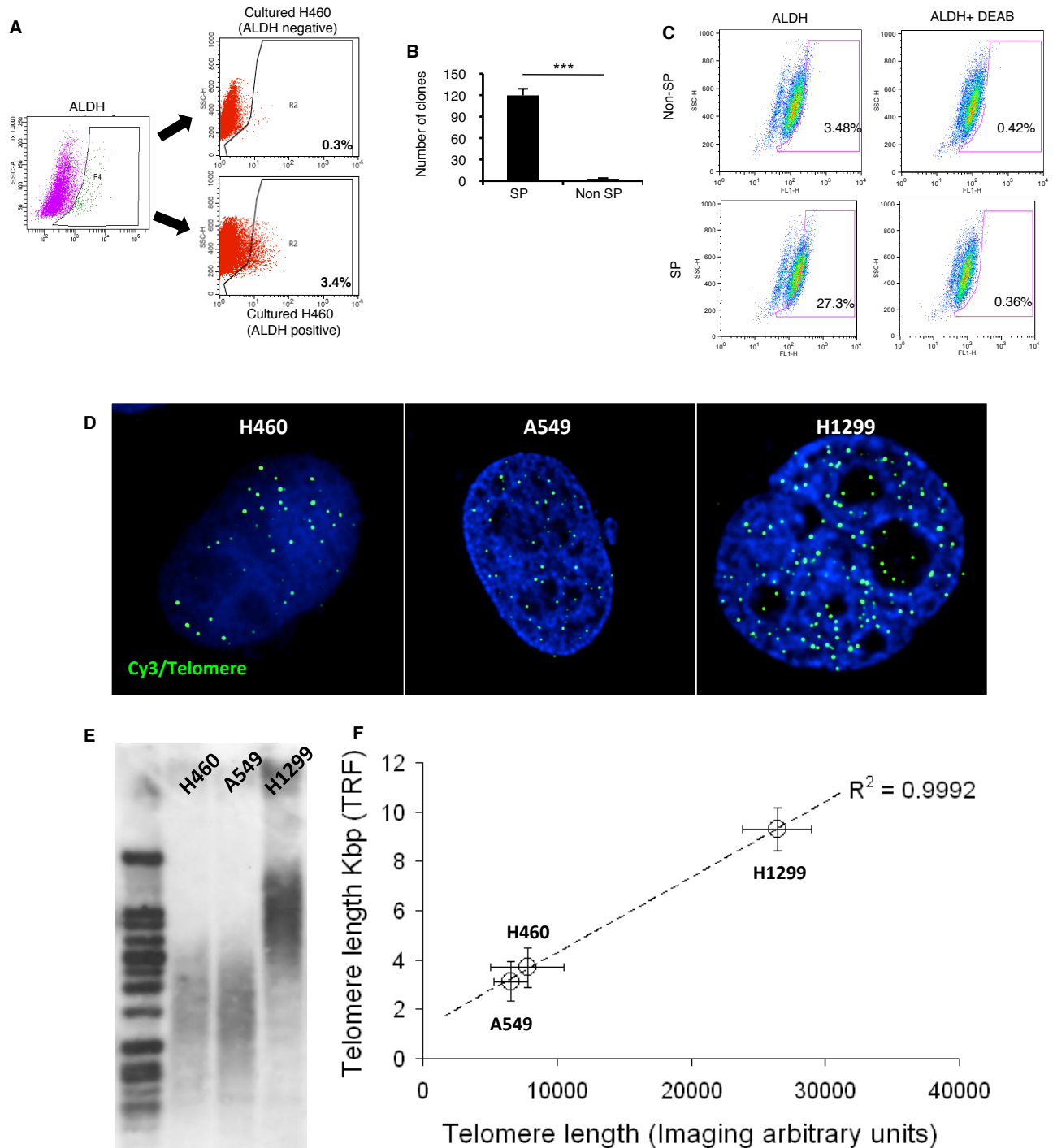

Supplement: Additional file 1 — Supplementary figure 1. A: Demonstration of the self-renewal capacity of ALDH+ H460 cells. Isolated ALDH+ cells give rise to both ALDH+ and ALDH- populations, while negative cells are not able to produce ALDH+ cells. B: Sorted H460 SP has significantly higher clonogenic capacity than non-SP cells. C: Demonstration that the H1299 SP overlaps with the ALDH+ populations. 27.3% of the SP corresponded to ALDH+ cells, whereas only 3.48% of non-SP were ALDH+. D: Representative FISH images from H460, A549 and H1299 unsorted cell lines used for the telomere quantification. E: Telomere length analysis by TRF; left, DNA ladder; right, H460, A549 and H1299 genomic DNA digested and hybridized with a telomeric specific probe. F: Correlation graph demonstrating the accuracy of extrapolation between image arbitrary units (FISH signals) and telomere length measured by TRF. ***: p < 0.001. [file 1476-4598-10-96-S1.PDF]

## Supplementary 2

Serrano et al.

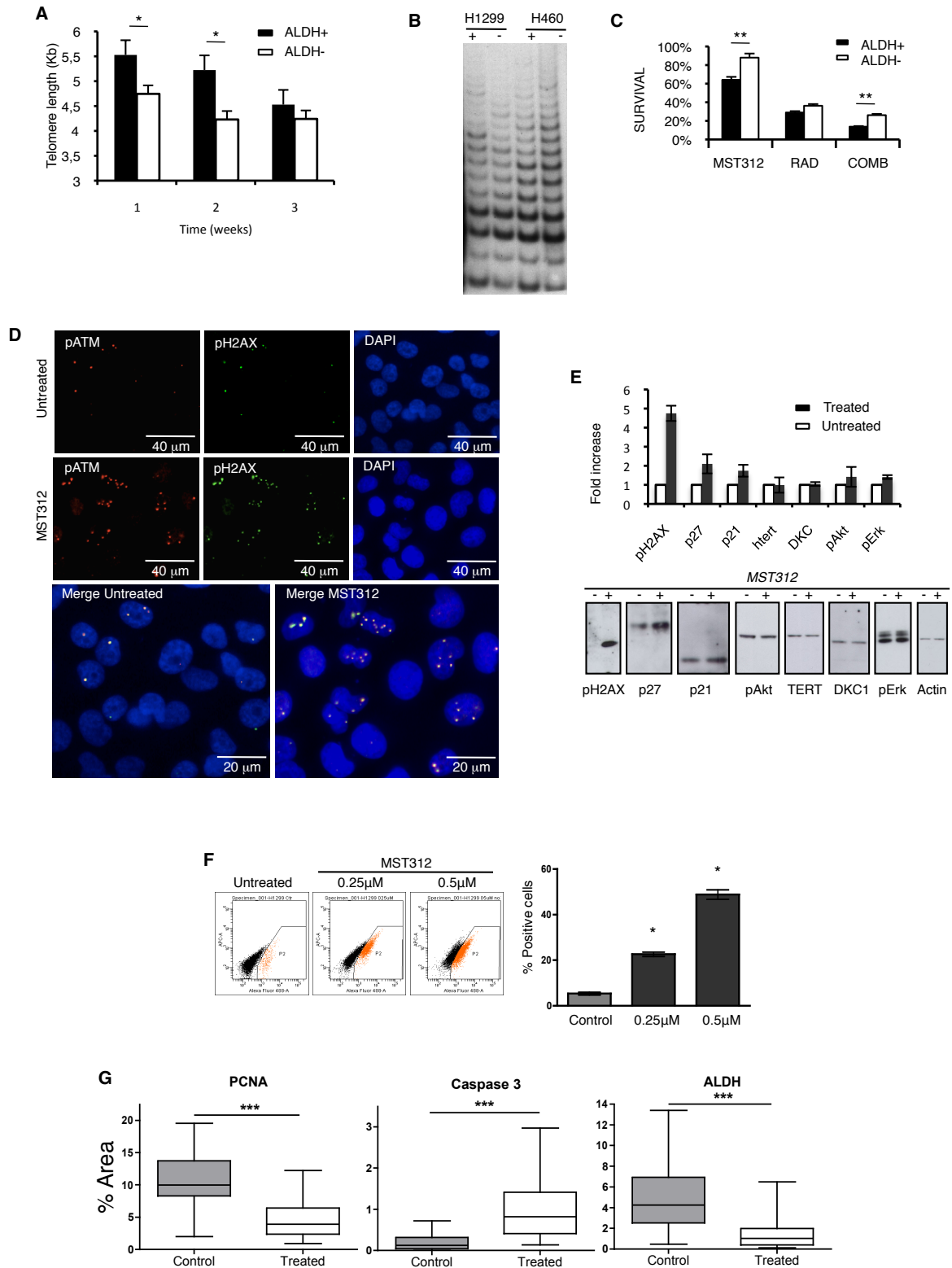

Supplement: Additional file 2 — Supplementary figure 2. A: ALDH+ and ALDH- cells were plated into separate culture slides and telomeric length was measured one and two weeks after plating. Telomeres in the ALDH+ population reached a similar length than that found in the negative fraction. This is likely due to the generation by ALDH+ cells of a majority of ALDH- population overtime, which supports the self-renewal capacity of these cells. B: Telomerase activity in both ALDH+/- cells. C: MST312 sensitizes ALDH+ cells to radiotherapy-induced cytotoxicity in vitro. Radiotherapy (RAD) alone causes a comparable decrease in cell survival of both ALDH positive and negative populations in H460 cells. Administration of MST312 sensitizes cells to irradiation-dependent cytotoxicity. D: Immunofluorescence for pATM (red) and pH2AX (green) in H460 cells, before and after MST312 treatment. An increase in both pATM and pH2AX (which co-localize) is detected as a result of the exposure to the telomerase inhibitor. E: Densitometric quantification of the bands from western blots of proteins related to cell cycle and DNA damage signaling cascades in untreated and MST312-treated cells. Three western blot analyses were quantified by densitometry; β-actin was used as housekeeping protein and data are presented in fold changes compared to the untreated group. Increase in pH2AX, p21 and p27 protein levels after 72 h incubation with MST312 was observed. No changes were found for hTERT, dyskerin (DKC1), pAkt and pErk. F: 10 days treatment with MST312 induces a dose-dependent increase in active caspase-3, H1299 cell line. G: Quantification of PCNA, cleaved-caspase 3 and ALDH staining by image analysis in tumor sections. *: p < 0.05; **: p < 0.01; ***: p < 0.001 compared with control. [file 1476-4598-10-96-S2.PDF]

Supplementary 3  
*Serrano et al.*

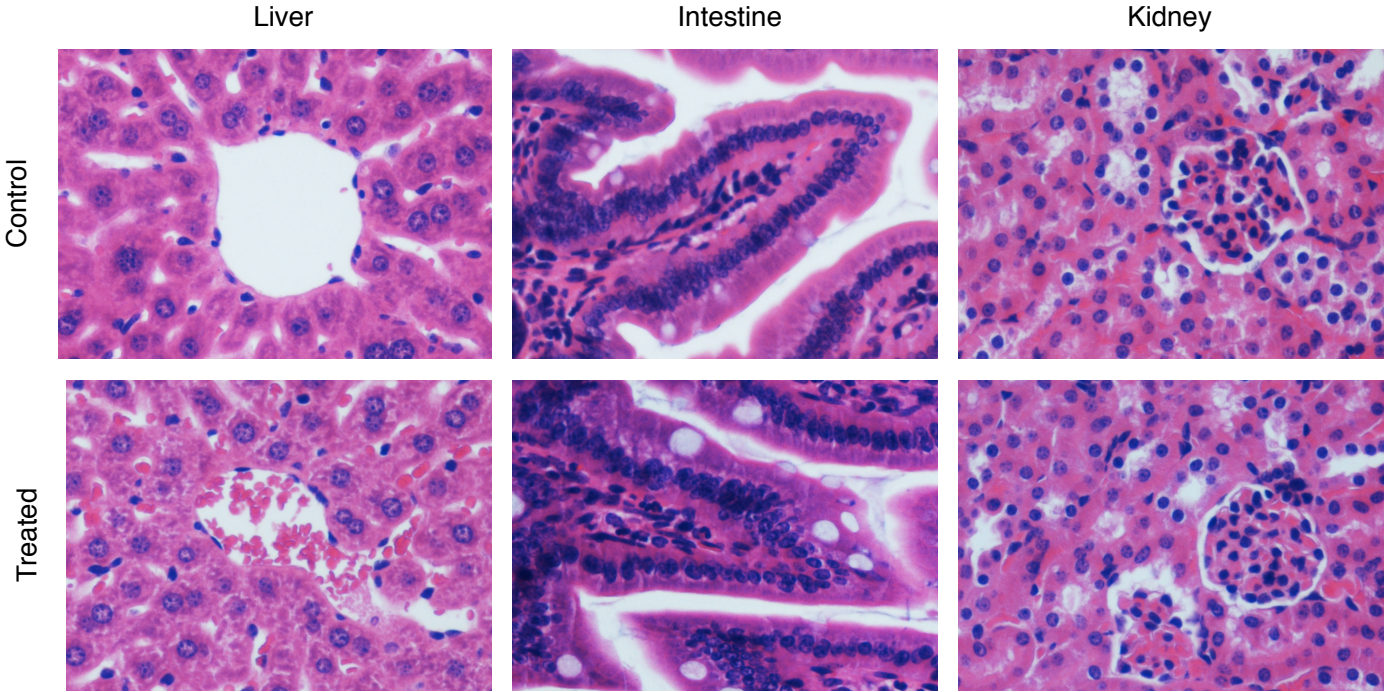

200X

Supplement: Additional file 3 — Supplementary figure 3. Histological appearance of liver, intestine and kidney in mice that received MST312 (treated) in comparison to controls. No significant changes are appreciated between both groups. [file 1476-4598-10-96-S3.PDF]
